# Editorial: New insights into the molecular mechanisms of autistic spectrum disorders

**DOI:** 10.3389/fnmol.2024.1363999

**Published:** 2024-01-22

**Authors:** Mojgan Rastegar

**Affiliations:** Department of Biochemistry and Medical Genetics, Max Rady College of Medicine, Rady Faculty of Health Sciences, University of Manitoba, Winnipeg, MB, Canada

**Keywords:** autism spectrum disorder, presynaptic proteins, gene editing techniques, CNTNAP2, amyloid-β precursor protein (APP) processing, Olink proteomics, MeCP2, SHANK3

The process of brain development is tightly controlled at different levels by genetics and epigenetics. Deregulation of gene regulatory mechanisms during development may lead to abnormal development and/or function of the brain that may be associated with autism spectrum disorders (ASD). The affected individuals may show a range of neurodevelopmental impairment with lifelong behavioral and social impact. This special topic includes eight articles, as a mini review, a manuscript in the category of hypothesis and theory, three review articles and three original research studies to highlight recent advancement in our knowledge about the underlying molecular mechanisms of ASD.

Focusing on gene editing technologies for monogenic ASD, Wang et al. discuss recent advances in genetic engineering that enables us to remove, inset, or modify a particular gene in cellular and animal model systems. The authors cover the involvement of MeCP2 and SHANK3 that have established links to mental disability and other neurodevelopmental disorders such as Rett Syndrome. Gene editing technologies are further illustrated with a detailed figure and multiple tables to provide a list of genes that have been subjected to gene editing technologies, animal model systems, and observed phenotypes. For specific gene editing technologies, the estimate cost, single or multiple gene editing techniques, off-target effect(s), and experimental timeline, as well as the pros and cons of each approach are highlighted. The authors cover certain mammalian and non-mammalian organisms for gene editing applications including *Drosophila, Zebrafish*, rodents, and non-human primates (Wang et al.).

The article by Yeo et al., focuses on the role of presynaptic proteins in ASD. In this review, the authors start with some historical description of autism, when it was first described over half of a century ago. They will then cover the developmental process of synaptogenesis with regards to presynaptic terminals and their functional role in neuronal characteristic and function. In this regard, the role of Liprin-α and Calcium/calmodulin dependent serine protein kinase CASK, as well as Synapsin, and other presynaptic proteins are detailed. Like the previous article, the authors use a comprehensive table and illustration to elegantly demonstrate the role/mutation of these proteins in connection with ASD, neuronal function, and clinical symptoms (Yeo et al.).

Complementing the second paper, Khoja et al., offer an interesting review on the presynaptic Neurexins focusing on Neurexin-2 in relation with ASD. The Neurexin family of proteins are encoded by three specific genes in mice (*Nrxn1, Nrxn2, Nrxn3*) and humans (*NRXN1, NRXN2, NRXN3*). The authors discuss the transcriptional regulation of these genes at the *cis* regulatory elements (promoter). This article also covers the associated mutations of the *NRXN* genes with ASD, and the role of Neurexins in controlling synaptic density and neuronal signal transmission. The authors further provide a table to highlight the brain regions where the role of Neurexins is studied in relation to transgenic animal models where these genes are manipulated and the impact in synaptic function. The authors also discuss the behavioral outcome of *Neurexin* gene mutation(s) in transgenic animal models (Khoja et al.).

A research article by Chen et al. presents an *in vitro* study in human cells that focuses on neuronal-specific epigenetic modification of histone H3 acetylation at specific genomic loci influenced by valproic acid. The authors use a series of cellular and molecular biology techniques to investigate the impact of valproic acid during the differentiation of human excitatory neurons. The authors conclude that valproic acid can block the proliferation of human neural progenitor cells without any significant impact on inhibitory neurons, while elevating neural progenitor cell differentiation into excitatory neurons (Chen et al.).

Another original research study by Zhang et al. focuses on the regulation of CNTNAP2 by hypoxia. In their studies, the authors implement a series of behavioral tests that would include the open field test, the Morris water maze, the social test, and Y maze test complemented by molecular biology experiments such as Western blot, 5′ RACE, and reporter assays. Their results highlight the impact of Oxygen homeostasis studied by induced hypoxia in the sociability and increased working memory of mice, as well as induction of CNTNAP2 in the murine brain. Further studies are completed *in vitro* in a human cell line to study the regulation of the *CNTNAP2* gene by determining the transcription start site by 5′ RACE and luciferase promoter assays. The connection of this study to ASD stems from the possible link between perinatal hypoxia to ASD, as indicated in the article (Zhang et al.).

As a hypothesis and theory contribution, Frackowiak and Mazur-Kolecka discuss the association of increased processing of amyloid-β precursor protein (APP) as a result of the function of particular secretases with that of autism spectrum disorders. APP is a sizeable transmembrane protein that is widely expressed and along with several other functions, it can stabilize the calcium fluxes in neurons. APP processing produces several fragments, that among them the Aβ peptides are of particular interest. The oligomeric Aβ peptides are suggested to have roles in retina development, that have led to the hypothesis of their involvement in embryonic neuronal development by the authors (Frackowiak and Mazur-Kolecka). This article covers a range of interesting topics about the Aβ peptides in relation to ASD, traumatic brain injury, and the pathophysiological outcome of the accumulation of Aβ in the brain, among others (Frackowiak and Mazur-Kolecka).

To continue of the connection of APP processing and Aβ with autism, Sokol and Lahiri contribute a review article. The authors describe how the increase in APP metabolites may have potential association with macrocephaly in autism spectrum disorders. The authors further highlight the properties of APP metabolites in quickening the growth of the glial cells and neurons of the brain. The authors link APP metabolites to specific signaling pathways that are detailed by illustrations. This article provides a clear summary of the current understanding on the association between APP metabolites and the potentially affected signaling pathways with that of ASD (Sokol and Lahiri).

Another original research on ASD is the study of Bao et al.. This article reports Olink proteomics in the plasma of 33 healthy individuals and 31 individuals diagnosed with ASD. The authors aim to investigate the alteration of plasma inflammation-related proteins in these samples. The authors identified 13 differentially expressed proteins with significant increase in ASD individuals compared to controls. Based on their findings, the authors conclude that inflammation may have important involvement in ASD and that increased level of related inflammatory proteins may have the potential as biomarkers for early diagnosis of ASD (Bao et al.).

## Author contributions

MR: Writing – original draft, Writing – review & editing.

